# Abl Kinase Inhibits the Engulfment of Apopotic Cells in Caenorhabditis elegans

**DOI:** 10.1371/journal.pbio.1000099

**Published:** 2009-04-28

**Authors:** Michael E Hurwitz, Pamela J Vanderzalm, Laird Bloom, Julia Goldman, Gian Garriga, H. Robert Horvitz

**Affiliations:** 1 Howard Hughes Medical Institute (HHMI), Department of Biology, MIT, Cambridge, Massachusetts, United States of America; 2 Massachusetts General Hospital Cancer Center, Boston, Massachusetts, United States of America; 3 Department of Molecular and Cell Biology, University of California, Berkeley, California, United States of America; 4 Helen Wills Neuroscience Institute, University of California, Berkeley, California, United States of America; St. Jude Children's Research Hospital, United States of America

## Abstract

The engulfment of apoptotic cells is required for normal metazoan development and tissue remodeling. In Caenorhabditis elegans, two parallel and partially redundant conserved pathways act in cell-corpse engulfment. One pathway includes the adaptor protein CED-2 CrkII and the small GTPase CED-10 Rac, and acts to rearrange the cytoskeleton of the engulfing cell. The other pathway includes the receptor tyrosine kinase CED-1 and might recruit membranes to extend the surface of the engulfing cell. Although many components required for engulfment have been identified, little is known about inhibition of engulfment. The tyrosine kinase Abl regulates the actin cytoskeleton in mammals and *Drosophila* in multiple ways. For example, Abl inhibits cell migration via phosphorylation of CrkII. We tested whether ABL-1, the C. elegans ortholog of Abl, inhibits the CED-2 CrkII-dependent engulfment of apoptotic cells. Our genetic studies indicate that ABL-1 inhibits apoptotic cell engulfment, but not through CED-2 CrkII, and instead acts in parallel to the two known engulfment pathways. The CED-10 Rac pathway is also required for proper migration of the distal tip cells (DTCs) during the development of the C. elegans gonad. The loss of ABL-1 function partially restores normal DTC migration in the CED-10 Rac pathway mutants. We found that ABI-1 the C. elegans homolog of mammalian Abi (Abl interactor) proteins, is required for engulfment of apoptotic cells and proper DTC migration. Like Abl, Abi proteins are cytoskeletal regulators. ABI-1 acts in parallel to the two known engulfment pathways, likely downstream of ABL-1. ABL-1 and ABI-1 interact physically in vitro. We propose that ABL-1 opposes the engulfment of apoptotic cells by inhibiting ABI-1 via a pathway that is distinct from the two known engulfment pathways.

## Author Summary

Cell death or apoptosis is a normal part of animal development, as is the engulfment and removal of dead cells by other cells. In the nematode Caenorhabditis elegans, ten highly conserved proteins have been characterized previously for their roles in engulfment and in cell migration, both of which involve the formation of cellular extensions. Little is known, however, about how engulfment is inhibited. In mammals, the tyrosine kinase Abl, which regulates the actin cytoskeleton and which when misexpressed causes two types of leukemia, prevents the CrkII protein from facilitating cell migration. CrkII functions in engulfment in C. elegans and mammals. We tested whether the C. elegans homolog of Abl, ABL-1, could inhibit engulfment. We found that ABL-1 functions as an inhibitor of apoptotic cell engulfment and cell migration. However, our analysis further showed that ABL-1 does not function by inhibiting other known engulfment proteins, including C. elegans CrkII. Our data indicate that ABL-1 blocks ABI-1, the C. elegans homolog of the mammalian and *Drosophila* Abl-interactor (Abi) cytoskeletal-regulatory proteins. We propose that ABL-1 acts via ABI-1 to inhibit a newly identified pathway during cell corpse engulfment and cell migration.

## Abstract 3

We show that*C. elegans* Abl (ABL-1) inhibits the engulfment of apoptotic cells via a newly defined pathway that includes the*C. elegans* homolog of the cytoskeletal regulator Abl-interactor.

## Introduction

**Figure 1:**
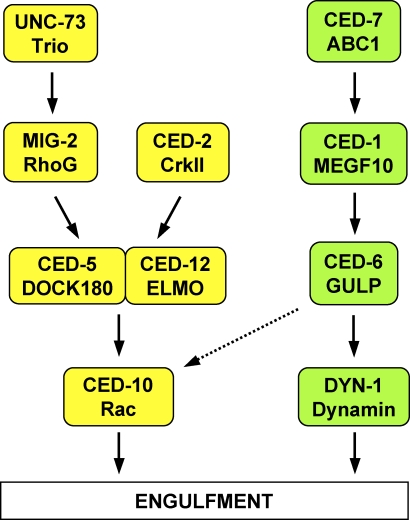
Molecular Pathways Required for the Engulfment of Apoptotic Cells Proteins of the CED-10 Rac pathway are labeled in yellow. Proteins of the CED-1 pathway are labeled in green. C. elegans protein names are written above and their mammalian homologs are below. The dashed arrow from CED-6 to CED-10 indicates that CED-1, CED-6, and CED-7 might also signal through CED-10 [19]. The CED-1 pathway is required for engulfment only, whereas the CED-10 Rac pathway is required for both engulfment and DTC migration. PSR-1 might act upstream of CED-2 [21].

Regulated reorganization of the cytoskeleton is a fundamental process in tissue morphogenesis and physiologic cell migration [1]. Dysregulation of the cytoskeleton is a hallmark of pathologic processes, such as cancer cell invasion and metastasis [2]. The engulfment of apoptotic cells requires a major cytoskeletal reorganization within the engulfing cell, which must extend its plasma membrane completely around the dying cell. In *C. elegans,* neighboring cells engulf apoptotic cells. Eleven genes appear to act in two parallel pathways required for engulfment: *ced-1, ced-6, ced-7*, and *dyn-1*; and *ced-2, ced-5, ced-10, ced-12, mig-2*, *unc-73*, and *psr-1* (Figure 1) [3]. These two pathways have been proposed to recruit membranes for cell surface extension and rearrange the cytoskeleton, respectively. The pathways together promote the extension of the engulfing cell around the apoptotic cell.

In the pathway for membrane recruitment, which we refer to as the CED-1 pathway (see below), four proteins have been identified (Figure 1). CED-7 is an ABC transporter required in both the engulfing cell and the engulfed cell and might expose a pro-engulfment signal on the surface of the apoptotic cell [4,5]. The role of CED-7 in the engulfing cell has not been defined. CED-7 is thought to signal through CED-1, a receptor on the engulfing cell surface homologous to *Drosophila* Draper and the mammalian EGF-like receptor MEGF10 [6]. CED-1, in turn, is proposed to signal through CED-6, a protein that contains a phosphotyrosine-binding domain [7]; CED-6 can bind a motif in the intracellular domain of CED-1 [8] and is thought to activate DYN-1, a C. elegans dynamin homolog [9]. DYN-1, by analogy to its role in vesicular transport in mammalian cells, might recruit membrane for the engulfment process. The CED-1 pathway also is involved in degrading apoptotic cells once they are engulfed [10,11].

The pathway for cytoskeletal rearrangement requires the small GTPase CED-10 Rac, and we refer to this pathway as the CED-10 Rac pathway. Two parallel pathways contribute to CED-10 Rac activation (Figure 1). CED-2, the C. elegans homolog of the oncoprotein CrkII, is an SH2 and SH3 domain-containing adaptor protein [12] that interacts with an atypical heterodimeric guanine nucleotide exchange factor (GEF) consisting of the proteins CED-5 [13] and CED-12 [14–16], homologs of mammalian DOCK180 and ELMO, respectively. In mammals, a signal from the apoptotic cell to the engulfing cell is transduced through CrkII to the DOCK180/ELMO heterodimer [17], and an analogous process is thought to occur between CED-2 and the CED-5/CED-12 heterodimer. The CED-5/CED-12 GEF activates the Rac1 homolog CED-10, and activated CED-10 rearranges the cytoskeleton [18,19]. Rac proteins are members of the Rho family of small GTPases that regulate the cytoskeleton and function in intracellular signaling [20]. The phosphatidylserine receptor PSR-1, which recognizes phosphatidylserine on the surface of the dying cell, has been proposed to act upstream of CED-2 [21].

MIG-2, the mammalian homolog of RhoG, another Rho family GTPase also regulates the CED-10 Rac pathway [22]. MIG-2 acts on CED-5/CED-12 in parallel to CED-2 [23]. UNC-73, a RhoGEF homologous to the mammalian protein Trio, activates MIG-2 [23]. The MIG-2 branch of the CED-10 Rac pathway provides a minor input into the engulfment pathway: mutations in *mig-2* and *unc-73* enhance the defects of other engulfment mutants but do not cause engulfment defects on their own [23].

Although many of the proteins that act in these two engulfment pathways are known, how these pathways are regulated is poorly understood. In mammals, the Abl tyrosine kinase functions in multiple processes that regulate the actin cytoskeleton [24]. For example, Abl blocks cell migration by phosphorylating and inhibiting the CED-2 homolog CrkII [25]. In addition to its role in Rac-dependent cell migration, Abl has been implicated in multiple signaling pathways in both the cytoplasm and the nucleus [26]. Abl acts in numerous cell biological processes, including cytoskeletal rearrangement, cell migration, apoptosis, transcription, and the response to oxidative stress [24,26]. Dysregulation of Abl via fusion of the *Abl* gene to the *BCR* gene is the cause of chronic myelogenous leukemia (CML) and an aggressive subtype of acute lymphoblastic leukemia (ALL) [27,28]. Recently, Abl signaling has been implicated in the prevention of breast cancer tumorigenesis [29] and in pathological fibrosis caused by the chemotherapeutic agent bleomycin [30,31].

That Abl functions in multiple diverse biological processes is reflected by its complex domain structure. In addition to its tyrosine kinase and DNA-binding domains, Abl has several protein-binding domains: Src Homology 2 (SH2), SH3, and F- and G-actin binding domains. Over 70 Abl-interacting proteins have been identified, largely through biochemical studies and cell-culture experiments. The in vivo relevance of most of these interactions has not been conclusively determined.

The C. elegans genome encodes a single Abl homolog, ABL-1. As in other organisms, Abl in C. elegans has many functions. ABL-1 protects germline cells from programmed cell death in response to ionizing radiation by antagonizing a molecular pathway that contains cell cycle checkpoint proteins and the p53 homolog CEP-1 [32]. ABL-1 is required for Shigella flexneri pathogenesis through an unknown mechanism [33]. A function for ABL-1 in cytoskeletal regulation has also been described: ABL-1 regulates epidermal morphogenesis in the C. elegans embryo by opposing the Ena/VASP homolog UNC-34 [34].

We found that ABL-1 inhibits the engulfment of apoptotic cells. Our genetic studies indicate that ABL-1 acts independently of both known engulfment pathways, suggesting the existence of another pathway for engulfment. We show that ABI-1, the C. elegans homolog of the Abi (Abl interactor) cytoskeletal and signaling family of proteins, is a member of this newly identified pathway.

## Results

### ABL-1 Inhibits the Engulfment of Apoptotic Cell Corpses

To test whether *abl-1* has a role in engulfment, we counted the number of unengulfed apoptotic cell corpses in the heads of first larval stage (L1) animals harboring mutations in *abl-1* and engulfment pathway genes. The number of unengulfed corpses varies with the strength of the engulfment defect and defines a quantitative assay of engulfment defects [35]. We used two presumptive null alleles of *abl-1* in this study, *n1963* and *ok171*. *n1963* is a G-to-A transition at the splice acceptor of exon 10 (bp 16967 of the M79 cosmid sequence), resulting in removal of most of the kinase domain and the change of a conserved arginine to serine. *ok171* is a deletion allele that removes the entire kinase domain, most of the SH2 domain, and results in a frameshift and an opal stop codon 52 bp later [32].

*abl-1* mutation alone had no obvious effect on engulfment. However, mutation of *abl-1* decreased the number of unengulfed corpses in the heads of *ced-1, ced-6*, and *ced-7* mutants (alleles *e1735*, *n2095*, and *n1892*, respectively, all of which are nulls) (Table 1)*. dyn-1* mutants die as embryos and were not tested. ABL-1 function did not depend on the presence of functional CED-1, CED-6, or CED-7 and therefore ABL-1 acts independently or downstream of the CED-1 pathway. These data are consistent with a role for ABL-1 in the negative regulation of apoptotic cell engulfment. Experiments that address alternative explanations for the affect of ABL-1 on engulfment are presented in the next section.

**Table 1:**
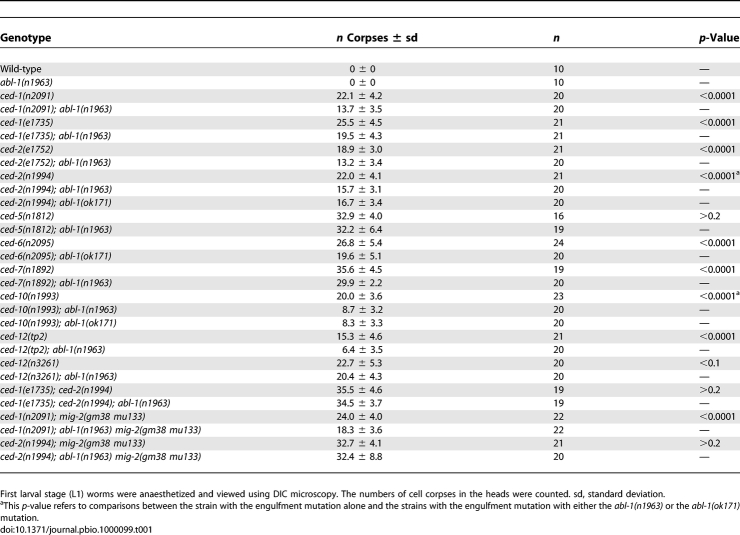
*abl-1* Mutations Suppress the Engulfment Defects of Engulfment *ced* Gene Mutations

We tested for interactions between *abl-1* and the genes of the CED-10 Rac pathway. *abl-1* mutation did not modify the engulfment defects of either *ced-5(n1812)* or *ced-12(n3261)* null mutants (Table 1). *abl-1* mutation also did not modify the engulfment defect of *ced-2(n1994); mig-2(gm38 mu133)* null double mutants, in which both known inputs into the CED-10 Rac pathway are absent (Figure 1). However, *abl-1* mutation did partially suppress the engulfment defect caused by *ced-2(n1994)* alone (the number of cell corpses decreased from 22.0 to 15.7, *p* < 0.0001) and by the partial loss-of-function allele *ced-2(e1752)* alone (a decrease from 18.9 to 13.2, *p* < 0.0001). *mig-2* null mutations do not cause engulfment defects on their own so they were tested in combination with a CED-1 pathway mutant (see below). Animals completely lacking *ced-10* die as embryos and were not tested, but the engulfment defect caused by a partial loss-of-function allele, *ced-10(n1993),* was suppressed by *abl-1*(lf) (the number of cell corpses decreased from 20 to 8.7, *p* < 0.0001).

We also tested whether the engulfment defect of a *ced-1(e1735); ced-2(n1994)* double mutant could be suppressed. A mutation in *abl-1* did not modify the engulfment defect of the double mutant, even though each single mutant was suppressed. This result is consistent with the possibility that ABL-1 acts upstream of both the CED-10 Rac and the CED-1 pathways. Alternatively, ABL-1 might suppress a pathway parallel to these two pathways, but its suppression might be too weak to modify an engulfment defect as severe as that of the *ced-1(e1735); ced-2(n1994)* double mutant. We present data below that support the latter model.

Because *mig-2* and *unc-73* mutations enhance the engulfment defects of other engulfment gene mutations but do not cause defects on their own [23], we tested whether ABL-1 acts through the MIG-2 branch of the CED-10 Rac pathway by testing whether an *abl-1* mutation could suppress the *mig-2* enhancement of *ced-1(n2091).* We observed fewer apoptotic cell corpses in the heads of *ced-1(n2091); mig-2(gm38 mu133)* animals when an *abl-1* mutation was present (Table 1), demonstrating that ABL-1 can act in the absence of MIG-2 function. Therefore, ABL-1 does not act solely through either the CED-2 branch or the MIG-2 branch of the CED-10 Rac engulfment pathway.

In summary, *abl-1*(lf) suppressed partial but not complete loss of the CED-10 Rac pathway. The inability of *abl-1*(lf) to suppress the engulfment defects of the CED-10 Rac pathway when this pathway was completely nonfunctional (i.e., *ced-5* null, *ced-12* null, or *ced-2; mig-2* double null mutants, Figure 1) suggests that the CED-10 Rac pathway genes do not function by blocking the action of ABL-1. Instead, ABL-1 might inhibit the CED-10 Rac pathway, or ABL-1 might signal in parallel to the CED-10 Rac pathway through another group of effectors that require CED-10 Rac pathway function to accomplish apoptotic cell engulfment.

Notably, the observation that *abl-1*(lf) suppressed the engulfment defect of mutants that completely lack CED-2 function indicates that the effect of ABL-1 on engulfment is at least partially independent of CED-2, i.e., ABL-1 does not act only by inhibiting CED-2. Therefore, C. elegans ABL-1 can act to inhibit CED-2 CrkII-dependent pathways via a mechanism distinct from the known mechanism in mammals, in which Abl phosphorylates the CED-2 homolog CrkII.

### ABL-1 Does Not Affect the Cell-Death Process Directly

The effect of *abl-1* mutation on the number of unengulfed corpses could be caused by mechanisms other than the direct inhibition of cell engulfment. *abl-1* mutation might (1) suppress programmed cell death, resulting in fewer cell corpses; (2) alter the timing of corpse appearance during development, resulting in fewer corpses at the time of observation; or (3) change cell-corpses so that they were not recognized as corpses or were unstable and were lost altogether.

To determine whether *abl-1* acts in programmed cell death, we evaluated whether cells known to undergo programmed cell death did so normally. In wild-type animals, 16 cells undergo programmed cell death in the anterior pharynx during embryogenesis [36], and their deaths can be scored by direct observation of their nuclei using DIC microscopy [35]. Mutants defective in programmed cell death, such as mutants with null mutations in the caspase *ced-3*, have up to 14 extra recognizable cell nuclei in the anterior pharynx [35,37]. We observed no extra nuclei in either *abl-1(n1963)* or *abl-1(ok171)* animals (Table 2). We also used a more stringent test for cell-death defects: enhancement of the death defect of *ced-3(n2427)* mutants, which are partially defective in programmed cell death [38] . As shown in Table 2, *ced-3(n2427)* animals had an average of 1.7 extra cells, and *abl-1* mutation did not enhance the c*ed-3(n2427)* death defect.

**Table 2:**
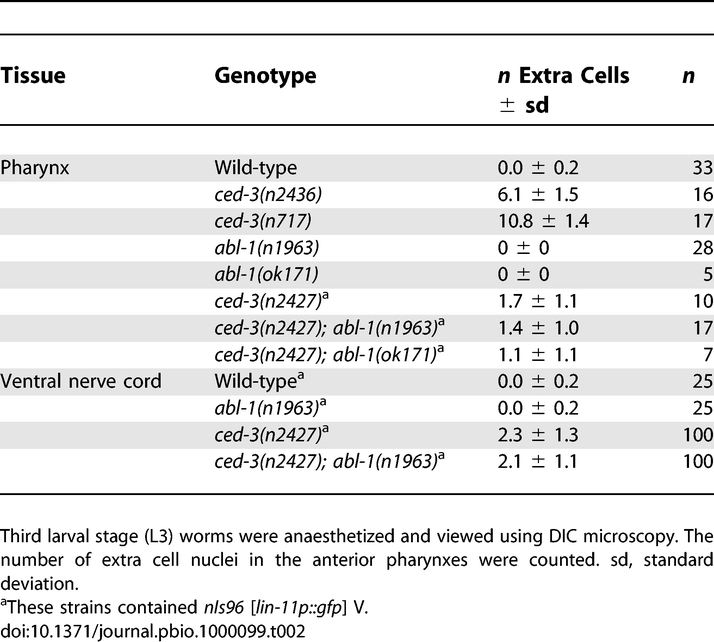
*abl-1* Mutation Does Not Block Cell Death

In the ventral nerve cord, six Pn.aap cells (P1.aap, P2.aap, and P9–12.aap) undergo programmed cell death postembryonically in wild-type animals, but not in death-deficient mutants [38,39]. Defects in programmed cell death are easily detected and quantified for five of these cells (P2.aap, P9-P12.aap) using a *lin-11::gfp* transcriptional reporter transgene, which is expressed by surviving Pn.aap cells [38]. As in the pharynx, in the ventral nerve cord *abl-1* mutation neither caused excess cell survival on its own nor enhanced the death defect caused by the *ced-3(n2427)* mutation (Table 2). We conclude that ABL-1 did not promote programmed cell death of non-germline cells. ABL-1 does protect against programmed cell death in the germline, most notably after radiation exposure [32]. In the germline *abl-1* loss-of-function therefore causes excess cell death, not suppression of cell death. If this function of ABL-1 were present in non-germline cells, we would expect increased numbers of corpses, not the reduction of unengulfed corpses that we observed.

**Figure 2:**
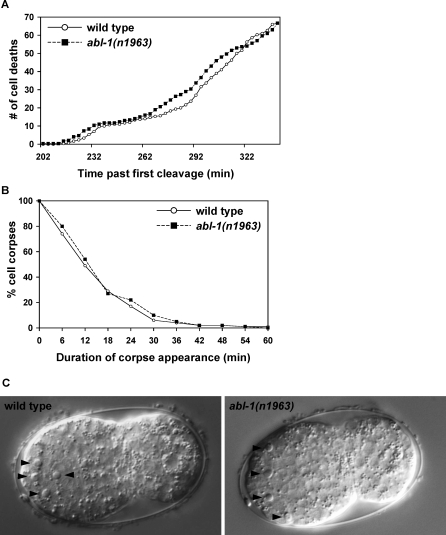
*abl-1* Mutation Does Not Affect the Timing or Morphology of Cell Corpses (A) The number and time of appearance of apoptotic cell corpses that occurred from 200–340 min after the first embryonic cell cleavage was recorded at 3-min intervals in wild-type and *abl-1(n1963)* animals using time-lapse DIC microscopy (see Materials and Methods). Mean numbers of corpses at each time point were calculated from three embryos for both wild-type and *abl-1(n1963)* animals. The curves are similar (*p* = 0.49). (B) The duration of cell-corpse appearance is similar in wild-type and *abl-1(n1963)* embryos. The percentage of cell corpses that lasted for a given period were recorded. The duration of appearance of all cell corpses recorded from three wild-type (*n =* 162 cell corpses) and three *abl-1(n1963)* (*n =* 171) embryos was analyzed. The curves are similar (*p* = 0.97). (C) The morphology of cell corpses in wild-type and *abl-1(n1963)* embryos are similar. Arrowheads, apoptotic corpses. Embryos were at a similar stage of development, approximately 300 min after the first cell corpse appeared.

To test whether ABL-1 affects the timing, persistence, or morphology of cell corpses, we used time-lapse DIC microscopy to observe wild-type and *abl-1(n1963)* embryos for 150 min after the first appearance of a cell corpse [9]. During this time, approximately 70 cell corpses appear in the wild-type animal. We observed no significant difference between wild-type animals and *abl-1* mutants with respect to the number of corpses that appeared or when they appeared (Figure 2A). Also, the length of time that corpses persisted was similar between wild-type and *abl-1(n1963)* animals (Figure 2B). In addition, apoptotic cell corpses in *abl-1(n1963)* animals looked identical to wild-type corpses (Figure 2C). We conclude that loss of ABL-1 did not affect the time of appearance or morphology of apoptotic cell corpses.

### *abl-1* Mutation Suppresses Other Defects Associated with Engulfment Pathway Genes

Mutants defective in corpse engulfment are also partially defective in programmed cell death, indicating that cell-corpse engulfment promotes cell killing [38,40]. A role for engulfment genes in promoting programmed cell death has also been found in *Drosophila* [41], showing that the pro-apoptotic function of cell engulfment is evolutionarily conserved.

In C. elegans mutants partially defective in cell-killing (e.g., *ced-3* caspase partial loss-of-function mutants), some cells that are fated to die undergo some of the morphological changes that accompany programmed cell death but then recover and persist as normal cells [38,40]. Most cells fated to die will nonetheless die. In C. elegans mutants with a partial loss of *ced-3* function and a mutation in an engulfment gene, a much larger number of cells fated to die will survive.

**Figure 3:**
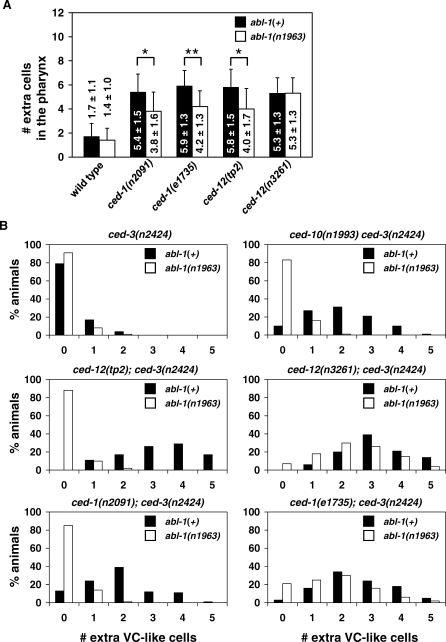
*abl-1* Suppresses the Cell-Killing Effect of Engulfment Pathway Genes Animals doubly mutant for *ced-3* and an engulfment gene with or without *abl-1(n1963)* were scored for the presence of extra cells in two tissues. (A) *abl-1* suppresses the engulfment gene cell-killing effect in the pharynx. Extra cell nuclei in the pharynges of animals in the early third larval stage (early L3) were counted using DIC microscopy. All animals harbored the *ced-3(n2427)* mutation. Means and standard deviations are shown. Error bars, standard deviation. At least ten animals were scored for each genotype. *, *p* < 0.005; **, *p* < 0.001. (B) *abl-1* suppresses the engulfment gene cell-killing effect in the ventral nerve cord. Extra GFP^+^ cells were counted in the ventral nerve cords of late fourth larval stage (L4) animals. All animals carried the *ced-3(n2424)* mutation and the insertion *nIs96*[*lin-11::gfp*], which labels VC neurons (P3–8.aap) and VC neuron-like cells (P1.aap, P2.aap, and P9–12.aap) that are normally fated to die. P1.aap is variably labeled by *nIs96*[*lin-11::gfp*] and was not scored. 100 animals of each genotype were scored. The number of extra VC-like cells were compared between strains containing *ced-3(n2424)* and a mutation in an engulfment gene with or without *abl-1(n1963)*. *p*-Values for differences between strains were as follows: for *ced-10(n1993)-*containing strains, *p* = 2.2 × 10^−16^; for *ced-1(n2091)-*containing strains, *p* = 4.4 × 10^−9^; for *ced-12(tp2)-*containing strains, *p* = 3.3 × 10^−8^. For all other strains, *p* > 0.7.

We tested whether *abl-1* mutations suppress the cell-death defect caused by engulfment gene mutations. The anterior pharynges of animals doubly mutant for *ced-3(n2427),* a partial loss-of-function mutation, and an engulfment gene mutation with or without *abl-1(n1963)* were scored for the presence of extra nuclei (Figure 3A). Partial loss-of-function and null mutations in *ced-1* and *ced-12* were analyzed. We observed fewer extra nuclei in animals that had the *abl-1(n1963)* mutation with either of the *ced-1* mutations and with the partial loss-of-function *ced-12(tp2)* mutation (Figure 3A). For example, *ced-1(e1735); ced-3(n2427)* animals had an average of 5.9 extra cells in their pharynges, whereas *ced-1(e1735); ced-3(n2427); abl-1(n1963)* animals had an average of 4.2 extra cells (*p* < 0.001). However, *abl-1* mutation did not affect the number of extra cells seen in *ced-12(n3261); ced-3(n2427)* animals. These findings are consistent with our observations concerning the role of ABL-1 in engulfment: *abl-1* mutation suppressed the death defect of a null mutation in the CED-1 pathway but did not suppress the death defect of a null mutation in the CED-10 Rac pathway (*ced-12(n3261))*.

We also examined the effect of an *abl-1* mutation on engulfment gene-related death defects in the ventral nerve cord using the *lin-11::gfp* transgene and the *n2424* partial loss-of-function allele of *ced-3*. As in the anterior pharynx, the death defects of weak alleles of *ced-1* and *ced-12* were suppressed by loss of *abl-1,* but a null *ced-12* allele was not strikingly suppressed (Figure 3B). Loss of *abl-1* also suppressed the death defect of the partial loss-of-function allele *ced-10(n1993)* in this assay. However, unlike what was seen in the pharynx, *abl-1* loss did not suppress the *ced-1* null defect appreciably in the ventral nerve cord (Figure 3A). While it is possible that this disparity is caused by differences in the role of *abl-1* in the ventral nerve cord and the pharynx, we prefer the hypothesis that the lack of suppression results from a relative insensitivity of the assay for ventral cord survival, i.e., loss of *abl-1* is insufficient to suppress the effect of the loss of *ced-1* function in this assay.

**Figure 4:**
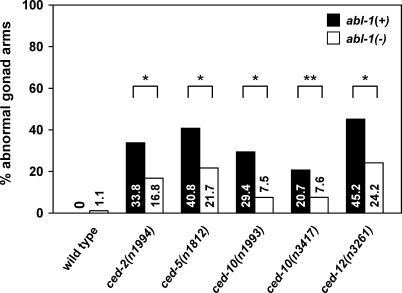
*abl-1* Mutation Suppresses the DTC Migration Defects of all CED-10 Rac Pathway Gene Mutations The gonads of animals mutant for an engulfment gene with or without *abl-1* mutation were observed and scored for morphology using DIC microscopy. Scoring was as described in Materials and Methods. Percentages of abnormal gonad arms are shown. At least 50 gonad arms were scored for the wild-type and *abl-1(n1963)* mutants*.* More than 100 gonad arms were scored for all other genotypes. All mutant *abl-1* strains used the *abl-1(n1963)* allele except for *ced-10(n3417),* which used *abl-1(ok171).* Statistical analysis used Fisher's exact test, *, *p* < 1x10^−5^; **, *p* < 0.005.

Mutants of the CED-10 Rac pathway have defects in cell migration. The two distal tip cells (DTCs) each migrate along a U-shaped trajectory during the development of the animal, guiding the formation of the gonads [42]. The gonads of CED-10 Rac pathway mutants often have an extra turn or have extra arms caused by abnormalities in DTC migration [43]. We tested whether *abl-1* mutation could decrease the percentage of gonadal abnormalities in CED-10 Rac pathway mutants. We observed no effect of *abl-1* mutation alone on DTC migration. Notably, *abl-1* mutation suppressed the gonadal morphology defects of all CED-10 Rac pathway mutants tested, including those caused by null *ced-5* and *ced-12* mutations (Figure 4). The percentage of defective gonadal arms in *ced-5(n1812)* animals decreased from 40.8% to 21.7% in *ced-5(n1812); abl-1(n1963)* animals (*p* < 1 × 10^5^), and the percentage in *ced-12(n3261)* animals decreased from 45.2% to 24.2% in *ced-12(n3261); abl-1(n1963)* animals (*p* < 1 × 10^5^). This analysis indicates that ABL-1 negatively regulates DTC migration and does not act through the genes of the CED-10 Rac pathway to do so.

We also examined the effect of a loss of *abl-1* function on the DTC migration defect of an animal harboring the *ced-10(n3417)* deletion mutation, a putative *ced-10* null allele [22]*.* Because the *ced-10(n3417)* mutation causes maternal-effect lethality (i.e., homozygous null animals produce no live progeny), we analyzed the homozygous progeny of *ced-10* heterozygotes (*ced-10(n3417)/lin-1(e1275) dpy-13(e184sd)*) with or without the *abl-1(ok171)* deletion mutation. These *ced-10(n3417)* homozygous animals presumably survived because they have CED-10 protein derived from maternally provided *ced-10* mRNA. As with *ced-2*, *ced-5*, and *ced-12* null mutants, the DTC defect of these *ced-10* null mutants was suppressed by an *abl-1* loss-of-function mutation: the percentage of defective gonadal arms in *ced-10(n3417)* animals decreased from 20.7% to 7.6% in *ced-10(n3417); abl-1(ok171)* animals (*p* < 0.005) (Figure 4)*.* We note that these *ced-10* animals are unlikely to totally lack *ced-10* function, since the *ced-10* null phenotype is maternal-effect lethal, indicating that *ced-10* homozygotes derived from *ced-10*/+ heterozygotes have some *ced-10* function; that *ced-10*/+ heterozygotes indeed have some *ced-10* function is supported by the observation that only 20.7% of the DTCs of the *ced-10(n3417)* animals migrated inappropriately, which is far less than that seen in *ced-5(n1812)*, *ced-12(n3261)*, or *ced-10(n1993)* animals. Therefore, no compelling conclusion about whether ABL-1 acts in parallel to or downstream of the CED-10 pathway can be made on the basis of this experiment.

Notably, *ced-10(n3417); abl-1(ok171)* animals produced a small number of live progeny (unlike *ced-10(n3417)* animals), some of which achieved adulthood; none of these was fertile. This suppression of the maternal-effect lethality caused by *ced-10(n3417)* might well reflect an effect of the *abl-1(ok171)* mutation in the complete absence of *ced-10* function. If so, at least in this case, *abl-1* acts in parallel to or downstream of *ced-10*. We suggest it is simplest to postulate that *abl-1* also acts in parallel to or downstream of *ced-10* for engulfment, engulfment-mediated programmed cell death, and DTC migration.

Our findings concerning engulfment and gonadal migration are consistent with two models of ABL-1 function. In one model, ABL-1 acts through different molecular pathways to inhibit the morphological changes that drive engulfment of apoptotic cells and to inhibit the migration of DTCs, i.e., ABL-1 acts directly on CED-10 or another protein in the CED-10 Rac pathway in engulfment and on a different set of proteins in DTC migration. Alternatively, ABL-1 acts in a pathway distinct from the CED-10 Rac pathway but common to both processes, and this common pathway is more important in DTC migration than in engulfment. For example, in gonadal cell migration, either the CED-10 Rac pathway or a second ABL-1*-*inhibited pathway might be sufficient for normal DTC migration. If this were the case, loss of ABL-1 function would derepress the ABL-1-regulated pathway and suppress DTC migration defects even in the absence of any CED-10 Rac pathway function, as we observed. In engulfment, the requirement for the CED-10 Rac pathway might not be able to be overcome by derepression of the ABL-1-regulated pathway. We present data below supporting the second model, namely that ABL-1 acts in a common pathway distinct from the CED-10 Rac and CED-1 pathways in engulfment and gonadal cell migration.

### ABL-1 Probably Acts in Engulfing Cells

To determine whether ABL-1 acts in the engulfing or the engulfed cell, we performed ectopic expression experiments in which *abl-1* was expressed from a transgene in an *abl-1* mutant background. Specifically, we used a protocol adapted from Reddien and Horvitz [12] and expressed *abl-1* under the control of C. elegans heat-shock promoters at a time at which all embryonic deaths are complete; we then counted the number of embryonic cell corpses in the heads of first larval stage (L1) animals. Any rescue that occurs cannot involve transgene function in the engulfed cells, which have already died. All cells in the head that die during embryogenesis do so prior to 5 h before hatching, and we scored engulfment within 5 h of heat shock. We found that expression of *abl-1* in *ced-10(n1993); abl-1(n1963)* animals increased the number of unengulfed corpses in L1 heads from 9.8 to 18.2 (Table 3) (for comparison, *ced-10[n1993]* animals had 20.0 corpses, Table 1). *abl-1* expression almost completely abrogated the effect of the *abl-1(n1963)* mutation, whereas expression of a *gfp*-only control transgene had minimal effects on the engulfment defect of *ced-10(n1993); abl-1(n1963)* animals (Table 3). In support of the idea that the engulfed cells did not make new proteins, GFP was not seen in the cell corpses (unpublished data). We concluded that *abl-1* is not required in the engulfed cell and therefore is likely required in the engulfing cell.

**Table 3:**
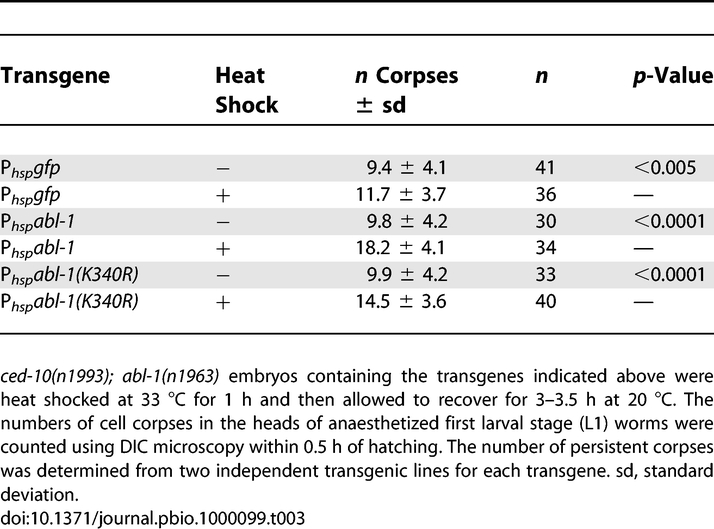
Overexpression of *abl-1* Reverses the Effect of *abl-1(n1963)* on Engulfment in *ced-10(n1993); abl-1(n1963)* Animals

We also tested whether ABL-1 kinase activity was required for *abl-1* reversal of engulfment suppression. We constructed an *abl-1* gene with a mutation that results in a change from lysine to arginine at position 340, *abl-1(K340R)*. Lysine 340 in ABL-1 corresponds to human c-Abl (isoform 1b) lysine 290, which when mutated disrupts Abl kinase activity [44–46]. We found that overexpression of *abl-1(K340R)* partially reversed the *abl-1(n1963)* suppression of engulfment in *ced-10(n1993); abl-1(n1963)* animals (Table 3). Although this result shows that the kinase activity of ABL-1 has a role in ABL-1–mediated engulfment suppression, it appears that ABL-1(K340R) might retain some activity. However, the result of this experiment must be interpreted cautiously. First, ABL-1(K340R) might have some residual kinase activity [47]. Even a small amount of residual kinase activity of ABL-1(K340R) after overexpression from an extrachromosomal array could result in partial reversal of the *abl-1(n1963)* phenotype. Moreover, high levels of ABL-1 protein might have nonphysiologic activity that allows ABL-1 to bypass the requirement for its kinase activity. Alternatively, the K340R substitution might destabilize the ABL-1 protein, resulting in lower ABL-1 protein levels, and a consequent decrease in the rescuing activity of the *abl-1(K340R)* transgene.

### ABI-1 Acts in Engulfment and DTC Migration

**Figure 5:**
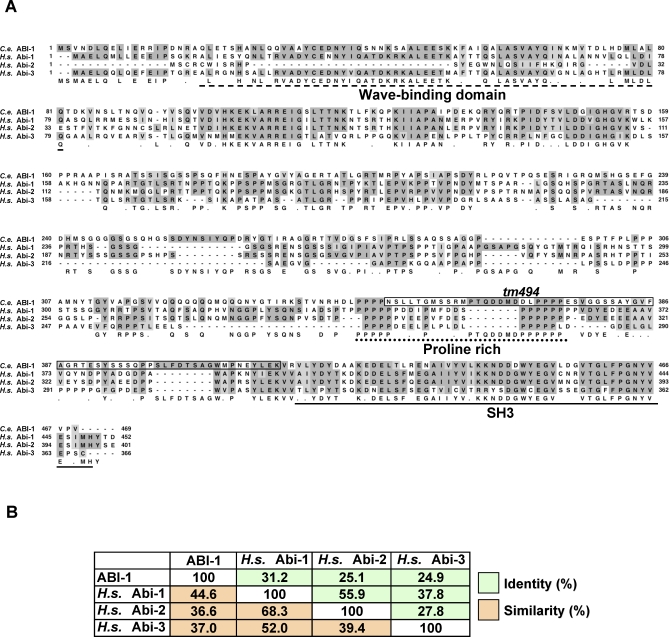
*abi-1* Is a *C. elegans Abi* Gene (A) Alignment of three Homo sapiens Abi proteins (Abi-1, Abi-2, and Abi-3) with C. elegans ABI-1. The dashed line indicates the Wave-binding domain. The dotted line shows the proline-rich region. The continuous line shows the SH3 domain. The boxed residues in ABI-1 indicate the sequence removed by the *tm494* deletion. Dark gray indicates identities, light gray similarities. (B) Similarity and identity indices between ABI-1 and each of the three human Abi proteins.

We tested whether ABI-1, the only C. elegans homolog of the Abi cytoskeletal regulatory gene family (Figure 5A), affects engulfment and gonadal cell migration. The Abi proteins (Abi-1/E3b1, Abi-2, and Abi-3/NESH in humans) were discovered as Abl interactors in yeast two-hybrid screens [48–50]. In different contexts, Abi and Abl proteins have been shown either to activate or suppress each other [48,49,51–57]. Abi proteins are part of the Scar/WAVE complex [58] and can interact with Formins [59], N-WASP [60], and Ena [52], all important regulators of the actin cytoskeleton. In addition, Abi proteins interact with signaling proteins that are important for cytoskeletal regulation, such as Eps8, Sos-1, and c-Cbl [50,55,61].

The Abi proteins have an N-terminal wave-binding domain [62], proline-rich repeats, and a C-terminal SH3 domain (Figure 5A). C. elegans ABI-1 is 31% identical to its closest human homolog, Abi-1 (Figure 5B), and has higher conservation in predicted functional domains. Our determination of *abi-1* transcript structures is described in Text S1.

We tested ABI-1 function using the deletion mutation *tm494*, which removes a 66-amino acid region just before the SH3 domain and changes the frame of the remaining sequence, resulting in a C-terminal truncation just before the SH3 domain. We also used *abi-1* RNAi by feeding to reduce *abi-1* expression [63,64], because RNAi by injection [65] causes embryonic lethality [66]. We showed that *abi-1(tm494)* and feeding *abi-1* RNAi probably caused a very weak loss of function, given that animals were viable and fertile. *abi-1(tm494)* or *abi-1* RNAi in wild-type animals caused a weak engulfment defect (Table 4). Also, the *tm494* mutation or *abi-1* RNAi significantly enhanced the engulfment defects of all engulfment mutants tested (in both the CED-10 Rac and CED-1 pathways) (Table 4). In addition, *abi-1* RNAi significantly enhanced the engulfment defect of a *ced-1(e1735); ced-5(n1812)* double mutant, in which both engulfment pathways are nonfunctional. To determine whether ABI-1 function requires ABL-1, we assayed the effect of *abi-1* RNAi in strains doubly mutant for engulfment genes and *abl-1*. Loss of *abi-1* function enhanced the engulfment defects of these strains to the same degree, whether *abl-1* mutation was present or absent. Therefore, ABI-1 does not act by modulating ABL-1 function.

**Table 4:**
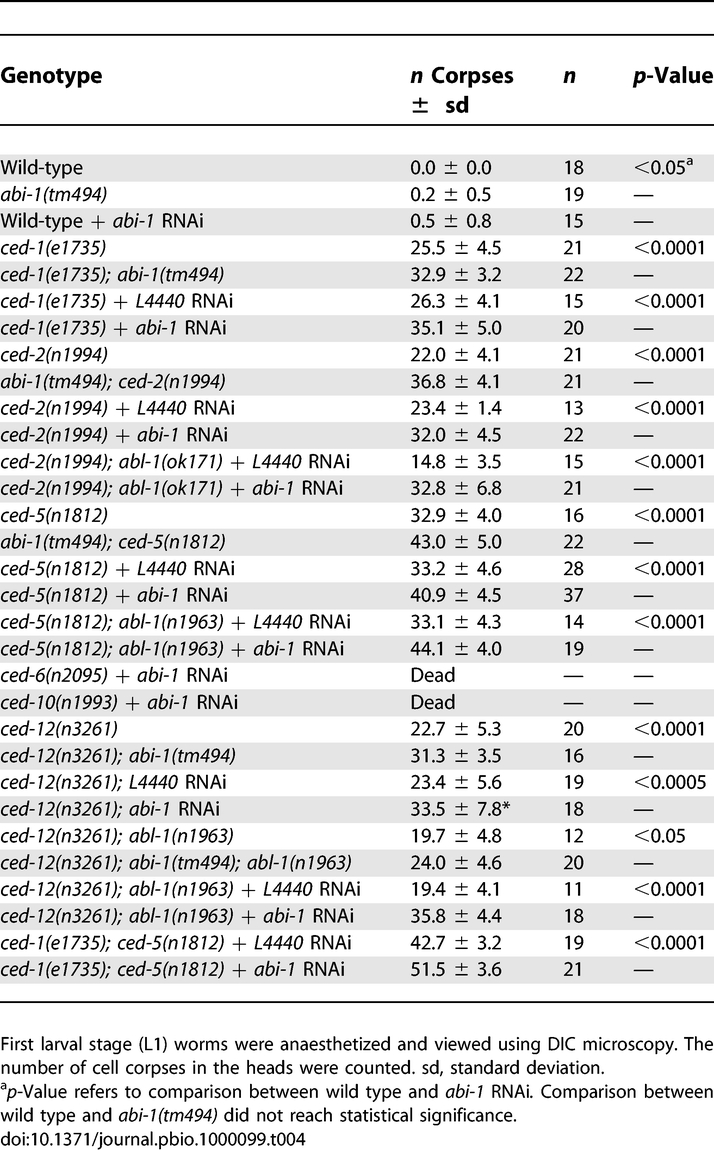
*abi-1* Mutation Enhances the Engulfment Defects of Engulfment Genes

**Figure 6:**
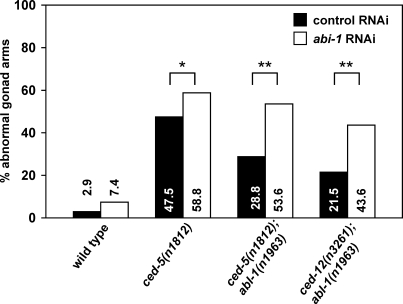
Loss of *abi-1* Function Enhances the DTC Migration Defects of Engulfment Pathway Genes The gonads of animals mutant for an engulfment gene with or without *abl-1(n1963)* were treated with *abi-1* RNAi or a control RNAi, observed, and scored for morphology using DIC microscopy. Scoring was as described in Materials and Methods. Percentages of abnormal gonad arms are shown. At least 50 gonad arms were scored for the wild-type and *abl-1(n1963)* mutants*.* More than 100 gonad arms were scored for all other genotypes. Statistical analysis used Fisher's exact test, *, *p* < 0.04; **, *p* < 1 × 10^−4^.

We also assayed the effect of *abi-1* RNAi on gonadal migration. *abi-1*RNAi enhanced the *ced-5* defect in gonadal cell migration (from 47.5% to 58.8%, *p* < 0.04) (Figure 6). Strikingly*, abi-1(*lf*)* completely abrogated the effect of *abl-1* mutation on gonadal cell migration in *ced-5* and *ced-12* animals.

Because Abi proteins are in the Scar/WAVE complex and other Scar/WAVE complex members have been implicated in engulfment (GEX-2 and GEX-3, the C. elegans homologs of Sra-1 and Nap, respectively) [67], we asked whether the localization of GEX-3 is altered in animals with a loss of *abl-1* function. We assessed the localization of a rescuing GFP::GEX-3 fusion protein (kindly provided by M. Soto) in embryos of animals that contain a *gex-3* null mutation *(zu196)* with either a wild-type *abl-1* allele or *abl-1(ok171)*. We found no localization of the fusion protein around embryonic cell corpses and no apparent difference in the pattern of fluorescence in both strains (Figure S1). Therefore, Scar/WAVE complexes do not appear to localize around apoptotic cell corpses at least at the time that corpses become visible by DIC optics. Furthermore, the absence of ABL-1 protein does not appear to alter the localization of Scar/WAVE complexes. However, ABL-1 probably only interacts with a fraction of the Scar/WAVE complexes present, and this fraction might not be sufficiently large to detect a difference in the overall localization of Scar/WAVE complexes using this method. Moreover, the rescuing GFP::GEX-3 fusion transgene is located on an extrachromosomal array and might be expressed at much higher levels than wild-type GEX-3. For these reasons, we cannot unambiguously interpret these results.

In summary, loss of ABI-1 function enhanced the engulfment defects caused by the inactivation of either the CED-10 Rac pathway or the CED-1 pathway or both together and enhanced the DTC migration defects of the CED-10 Rac pathway. ABI-1 action was not modified by ABL-1 inactivation. These data suggest that ABL-1 acts through ABI-1 to inhibit engulfment and DTC migration.

*abl-1* is expressed broadly throughout embryogenesis [32]. To determine where *abi-1* is expressed, we created an *abi-1::gfp* reporter transgene and observed expression of the *abi-1* reporter broadly throughout embryogenesis (unpublished data).

### ABL-1 and ABI-1 Interact In Vitro

**Figure 7:**
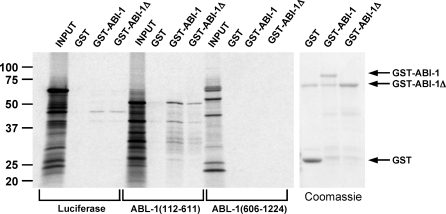
ABL-1 and ABI-1 Interact In Vitro GST, GST-ABI-1, and GST-ABIΔ were expressed, bound to glutathione beads, and incubated with in vitro translated Luciferase and portions of ABL-1: ABL-1(112–611) and ABL-1(606–1,224); numbers in parentheses represent amino acid positions. Beads were washed, diluted in sample buffer, and separated on SDS-PAGE. The gel was stained with Coomassie Blue, dried, and exposed. Left panel, autoradiograph. Input lanes contain 2% of each in vitro translation reaction. 25% of each in vitro translation reaction was used per binding experiment. GST, GST-ABI, and GST-ABIΔ lanes contain 25% of each binding experiment. Size markers in kilodaltons are shown on the left. Right panel, Coomassie Blue-stained gel showing relative amounts of GST, GST-ABI, and GST-ABIΔ used in each binding experiment. There is a nonspecific band that comigrates with GST-ABIΔ.

In mammals, Abl and Abi-2 interact in vitro in two ways. The SH3 domain of Abl binds to a site in the first 157 amino acids of Abi-2, likely a proline-rich site. The SH3 domain of Abi-2 binds to a proline-rich region near the center of Abl (amino acids 593–730) [49]. To test whether C. elegans ABL-1 and ABI-1 interact directly, we performed in vitro binding experiments with glutathione-S-transferase fused to the N terminus of ABI-1 and in vitro translated portions of ABL-1. We made two ABL-1 constructs, an N-terminal fragment (amino acids 112–611) and a C-terminal fragment (amino acids 606–1,224). ABL-1(112–611) bound to ABI-1, but to a small degree also bound to GST alone. Quantitation of the bands using phosphorimagery revealed 8-fold higher binding in the ABI-1 lane than in the GST lane despite a much smaller amount of ABI-1 than GST loaded on the gel (note Coomassie Blue-stained gel next to the autoradiograph) (Figure 7). ABI-1 did not bind to the control Luciferase; we also failed to observe ABL-1(606–1,224) binding. ABL-1(112–611) contains the SH3, SH2, and tyrosine kinase domains of ABL-1. ABL-1(606–1,224) contains the entire C-terminal half of ABL-1, including the region homologous to mammalian Abl where the Abi-2 SH3 domain binds. However, the polyproline stretch is within the first 60 amino acids of ABL-1(606–1,224) and might not have folded appropriately to bind to the ABI-1 SH3 domain. The direct binding of the N-terminal half of ABL-1 to ABI-1 strengthens the hypothesis that a direct interaction between these proteins exists in vivo and suggests that ABL-1 directly inhibits ABI-1 in its roles in the engulfment of apoptotic cells and DTC migration.

We also tested whether ABL-1 binds to the product of the *abi-1(tm494)* allele, which contains only the first 350 amino acids of the 470 amino acid protein. We called this protein ABI-1Δ. As described above, we made an N-terminal GST fusion to ABI-1Δ and tested whether it bound to ABL-1. Like ABI-1, ABI-1Δ bound to ABL-1(112–611) but not to ABL-1(606–1,224) or to Luciferase (Figure 7). This result is consistent with the fact that the region of ABI-1 thought to bind to the N terminus of ABL-1 on the basis of mammalian Abl/Abi interactions is intact in ABI-1Δ [49]. Although the amount of ABL-1(112–611) bound by ABI-1Δ was approximately 3-fold less than that bound by ABI-1, it is difficult to know how much ABI-1Δ was loaded onto the gel, because ABI-1Δ comigrates with a nonspecific band. Alternatively, truncation of ABI-1 might cause changes in folding that decrease its ability to bind substrates, which might reflect decreased binding to ABL-1 in vivo and possibly explain the decreased function of the *abi-1(tm494)* allele.

## Discussion

We have demonstrated that the C. elegans Abl ortholog ABL-1 negatively regulates the engulfment of apoptotic cells. *abl-1* inhibits the engulfment process as well as the engulfment-related cell-killing process and the migration of DTCs during gonadogenesis. Our genetic analysis suggests that ABL-1 acts in a manner that does not require the known engulfment pathways. Ectopic expression experiments indicate that ABL-1 acts in engulfing cells and that its function at least partially depends on its kinase activity. Moreover, our studies show that the Abi ortholog ABI-1 acts in engulfment and DTC migration. Finally, our genetic and biochemical studies both suggest that ABL-1 directly inhibits ABI-1 in a pathway distinct from the known engulfment gene pathways.

### How Do ABL-1 and ABI-1 Interact In Vivo?

Our genetic analysis indicates that either *abl-1* could inhibit *abi-1* or the two genes could act in separate molecular pathways. We favor the former hypothesis, both because ABL-1 and ABI-1 interact directly in vitro and because in mammalian cell culture and cultured neurons the homologs of these proteins interact and function in processes that regulate the cytoskeleton [53,56].

Our findings establish that an Abl protein and an Abi protein interact functionally in vivo. In some in vitro studies Abi activated Abl [49,51–54], in others Abl activated Abi [56,57], and in still others Abl appeared to block Abi function [48–50,55]. We found that *abl-1* and *abi-1* have opposing functions in vivo. Given the large number of proteins with which Abl and Abi interact and the multiple cellular contexts in which they function, in vivo analyses will be critical to distinguish which interactions are relevant for a particular cell biological process.

### ABL-1/ABI-1 Likely Act in Parallel to the CED-10 Rac and CED-1 Pathways

The inhibition of engulfment by ABL-1 occurred in the absence of functional CED-2 CrkII, indicating that the effect of ABL-1 on engulfment and DTC migration is not mediated by CED-2 CrkII inhibition. In mammals, Abl phosphorylates tyrosine 221 of CrkII between its SH3 domains, resulting in inhibition of CrkII function and suppression of cell migration [25,68]. This tyrosine is not conserved in *C. elegans.* We conclude that C. elegans ABL-1 blocks the CED-10 Rac pathway by a novel mechanism.

Our analysis of genetic interactions between *abl-1* and the engulfment genes suggests the existence of a new pathway involved in both cell-corpse engulfment and DTC migration. Loss of ABL-1 function suppressed the engulfment and cell-death defects of all CED-1 pathway genes tested. *abl-1* mutation did not suppress the engulfment defects of *ced-5* or *ced-12* nulls but did suppress their DTC migration defects. Since *abl-1* mutation modulated DTC migration in the absence of *ced-5* or *ced-12* function (i.e., when the CED-10 Rac pathway was inactive), ABL-1 can signal through another pathway. We propose that ABL-1 acts in a third pathway not only for DTC migration but also for engulfment. If so, this pathway cannot promote engulfment in the absence of CED-10 Rac activation. Although it is formally possible that the function of ABL-1 in engulfment is mediated through the CED-10 Rac pathway while its effect on DTC migration is mediated through a different pathway, we prefer a simpler model in which ABL-1 acts through a single pathway to oppose the engulfment genes. This pathway might act in parallel to CED-10 or it might act on CED-10 Rac (see below).

We found that ABI-1 is required for the function of ABL-1 in engulfment and DTC migration. ABI-1 promoted engulfment and migration independently of the known engulfment pathways downstream of or in parallel to ABL-1. We propose that ABL-1 inhibits ABI-1 and that these two proteins define a new molecular pathway required for cell-corpse engulfment and DTC migration. Interestingly, loss of *abl-1* did not suppress the engulfment defects of CED-10 Rac pathway null mutants, whereas loss of *abi-1* did enhance those engulfment defects. At least three models can explain these findings. ABL-1 might not be a sufficiently potent inhibitor of ABI-1 to affect engulfment in the absence of CED-10 Rac pathway activity. Second, the CED-10 Rac pathway might be absolutely required for engulfment, so that derepressing the ABL-1/ABI-1 pathway does not suppress the CED-10 Rac pathway engulfment defect. Third, loss of *abl-1* function might increase engulfment activity in the absence of the CED-10 Rac pathway insufficiently to detect in the engulfment assay.

Loss of *abl-1* function suppressed the DTC migration defect of a null *ced-10* mutant. However, these animals were the progeny of heterozygotes and probably contained some functional CED-10 protein. Loss of *abl-1* function suppressed the maternal-effect lethality of a *ced-10* null mutation. This observation provides stronger evidence that *abl-1* can act in parallel to or downstream of *ced-10*. We were unable to test the effects of *abl-1* or *abi-1* mutations on a null mutant of *dyn-1,* because this mutant arrests development during embryogenesis and, unlike in the case of a null *ced-10* mutant, DTC migration is not affected in *dyn-1* mutants. Therefore, we do not know whether ABL-1 or ABI-1 can act independently of CED-10 or DYN-1 for engulfment or engulfment-mediated programmed cell death (or DTC migration in the case of CED-10) and hence whether ABL-1 and ABI-1 act through either of these genes or in a parallel molecular pathway. We think it unlikely that ABL-1 and ABI-1 act through DYN-1*,* since the CED-1 pathway has no known role in DTC migration, and ABL-1 and ABI-1 modulate DTC migration defects.

**Figure 8:**
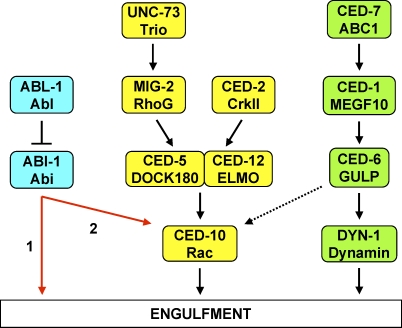
ABL-1 and ABI-1 Likely Function in Parallel to the CED-10 Rac and CED-1 Engulfment Pathways We suggest that ABL-1 inhibits ABI-1, which acts to promote the engulfment of apoptotic cells. ABI-1 might signal either independently of the CED-10 Rac pathway (Arrow 1) or through CED-10 Rac, in parallel to the CED-10 GEF CED-5/CED-12 (Arrow 2). This model also applies to the roles of ABL-1 and ABI-1 in the regulation of DTC migration, but we do not show DTC migration in the figure, because the CED-1 pathway does not act in this process. Since ABL-1 and ABI-1 act in DTC migration, ABL-1/ABI-1 cannot act solely through the CED-1 pathway since the proteins of the CED-1 pathway have no role in DTC migration.

There are at least three models for how the ABL-1 and ABI-1 proteins act in engulfment and DTC migration. First, ABL-1 might directly inhibit ABI-1 from promoting engulfment of apoptotic cells and inappropriate DTC migration through a molecular pathway that acts in parallel to the known engulfment gene pathways (Figure 8). Second, ABI-1 might act on CED-10 Rac in parallel to the CED-5/CED-12 heterodimer (Figure 8). These models are not mutually exclusive. In a third model, the CED-10 Rac pathway and ABL-1 both act on ABI-1 in parallel to each other, with CED-10 Rac activating ABI-1 and ABL-1 inhibiting it. Studies of mammalian Abi proteins are consistent with the first two models. For example, Abi proteins are found in complexes with N-WASP [60] and the Formins [59]. Both N-WASP and formins act in actin cytoskeletal rearrangements independently of the CED-10 homolog Rac. C. elegans ABI-1 might act similarly in our first model. Abi proteins also form a complex with Eps8 and Sos-1. Formation of the Abi-1/Eps8/Sos-1 complex activates the RacGEF activity of Sos-1 in response to tyrosine kinase signaling [50,61]. ABI-1 might act this way in our second model. In mammals, Rac and Abl proteins both act on the Scar/WAVE complex through interactions with Abi proteins [53,69]. However, in these cases Abl activates Abi. By contrast, we found that ABL-1 inhibits ABI-1. For this reason we do not favor a model in which ABL-1 and CED-10 Rac act on ABI-1 in parallel.

### Why Negatively Regulate Engulfment?

Despite the large number of genes known to be involved in engulfment, only a few negatively regulate the process. Loss of SWAN-1, a CED-10-binding protein, suppresses the engulfment and DTC migration defects caused by *ced-10* loss of function, and SWAN-1 thus might be a negative regulator of these processes [70]. In mammalian macrophages and macrophage cell lines, the small GTPase Rho and one of its effectors, Rho-kinase, negatively regulate engulfment of apoptotic cells [71,72]. Rho, like Rac, regulates the cytoskeleton, and in many contexts the two proteins act in opposition to each other [20]. All Rac proteins are downregulated by Rac-specific GTPases (RacGAPs) [73]. No RacGAPs have been identified that function in engulfment, though presumably one or more will be found.

Pathways that inhibit engulfment might prevent the inappropriate engulfment of healthy cells that are not programmed to die. There are examples of inappropriate engulfment of mammalian cells. In entosis, cells engulf and eventually kill neighboring cells that have lost their attachments to the extracellular matrix [74]. Also, the glycosylated surface protein SIRPα is found on engulfing cells and interacts with the integrin-associated protein CD47 on other viable cells. When that interaction is disrupted, inappropriate engulfment occurs [75]. Notably, the intracellular cascades that transduce these signaling events have not been discovered. Possibly *abl-1* transduces these types of signals.

Perhaps there are conditions that cause cells to be particularly sensitive to engulfment, and without such negative regulatory pathways cells would be inappropriately killed. Since engulfment promotes the cell-killing process, several of the situations in which engulfment occurs could be severely affected by such excess cell death: organismal development, wound healing and infection control.

Targeting signaling pathways that negatively regulate engulfment might have therapeutic benefits. For example, inducing professional engulfing cells, such as macrophages, to engulf diseased cells, such as cancerous cells or those infected with viruses or bacteria, could aid in combating these disease processes. Also, in humans impaired engulfment of apoptotic cells has been associated with systemic lupus erthematosus (SLE) [76] and in mice, ineffective engulfment of apoptotic cells can cause an SLE-like syndrome [77]. Enhancing the engulfment of apoptotic cells might aid in treating or preventing certain autoimmune disorders. Extremely effective and specific small molecule inhibitors of Abl, such as Imatinib [78] (Gleevec) and Nilotinib [79], exist so this idea could be tested.

## Materials and Methods

### Strains and genetics.

C. elegans strains were maintained at 20 °C as described [80]. The N2 Bristol strain was used as the wild-type strain. Animals were grown on NGM plates and fed OP50 bacteria [42]. The mutations and integrants used were: LGI, *ced-1(e1735, n2091), ced-12(n3261, tp2)*; LGIII, *abi-1(tm494), ced-6(n1813, n2095), ced-7(n1892)*; LGIV, *ced-2(e1752, n1994), ced-3(n717, n2424, n2427, n2436), ced-5(n1812), ced-10(n1993, n3417), dpy-13(e184sd), gex-3(zu1963), lin-1(e1275), unc-24(e138)*; LGV, *unc-34(e566, gm114), unc-76(e911), nIs96* [38]; LGX, *abl-1(gm327, gm332, n1961, n1963, n1964* [all this study; see below], *ok171), mig-2(gm38 mu133)*, *nIs106* [38]. Mutant alleles for which no citation is given were described previously [81]. Information about *ok* and *tm* alleles (*tm* alleles kindly provided by S. Mitani, Tokyo Women's Medical University, Japan) can be found at www.wormbase.org. The following balancer chromosomes were used: LGI; LGIII, *hT2[qIs48]*; LGII, *mIn1[mIs14]*; LGIV; LGV, *nT1[qIs51].*

### Isolation of *abl-1* alleles.

*abl-1* alleles *(gm327*, *gm332*, *n1961*, *n1963*, *n1964)* were isolated in two separate genetic screens for mutations that suppressed the Unc locomotion phenotype of *unc-34* mutants. *unc-34* mutant C. elegans were mutagenized with ethyl methanesulfonate (EMS) as described [80]. The F_2_ progeny of mutagenized *unc-34(e566)* hermaphrodites representing 17,500 haploid genomes were inspected for the presence of animals with normal locomotion. Three strains were identified as containing candidate suppressors *(n1961*, *n1963*, *n1964)* [82]. *gm327* and *gm332* were isolated in an F_1_ clonal screen. *unc-34(gm114)* P_0_ hermaphrodites were mutagenized and allowed to lay eggs. Single F_1_ animals were transferred onto fresh plates, and F_2_ progeny were screened for a non-Unc phenotype. 10,952 haploid genomes were screened this way.

In addition to the five alleles of *abl-1*, five other mutations were isolated in the above screens. Complementation and mapping studies demonstrated that these five mutations defined a second complementation group and are alleles of the gene *crml-1* [83]. See Text S1 for a more detailed description of the complementation tests and mapping and the cloning of the *abl-1* gene*.*

### Quantitation of engulfment defects.

Unengulfed apoptotic corpses were visualized in the heads of young larvae as refractile discs directly using Nomarski differential interference contrast (DIC) microscopy [39,84]. Apoptotic cell corpses were counted in the heads of first larval stage (L1) animals within 30 min of hatching, except for animals treated with RNAi (see below). Animals were anaesthetized in 30 mM sodium azide in M9 [42] and viewed using DIC optics on a Zeiss Axioskop 2 compound microscope (Thornwood). For animals treated with feeding RNAi, L1 animals were picked, and those with gonads that had not passed the four-cell stage were viewed as described above. *P*-values for pairwise comparisons were calculated using the Student's *t*-test.

### Quantitation of cell-death defects.

For quantitation of cell-death defects in the anterior pharynx, animals in the third larval stage (L3) were anaesthetized and viewed with DIC microscopy as described above. Briefly, the locations of the nuclei of the 16 cells that undergo programmed cell death in the anterior pharynx are known [36]. In wild-type animals by the L3 stage, all of those nuclei have disappeared; any remaining nuclei in the animals tested were scored as extra cells. *p*-Values for pairwise comparisons in the pharynges were calculated using Student's *t*-test. For quantitation of cell-death defects in the ventral nerve cord, a dissecting microscope equipped with an ultraviolet light source (Kramer Scientific) was used. To analyze ventral nerve cord engulfment, we used a Wilcoxon rank-sum test. For pairwise comparisons between strains with wild-type *abl-1* and mutant *abl-1*, we tested the null hypothesis that the median number of extra VC neuron-like cells in the wild-type strain was less than or equal to one more than the median number of VC neuron-like cells in the *abl-1* mutant strain. Tests were conducted with the wilcox.test function of R (www.r-project.org).

### Time-lapse microscopy.

Gravid C. elegans were dissected, and embryos at the two-cell stage were placed at 20 °C for 180 min. Single embryos were then placed on an agar pad, sealed with petroleum jelly, and viewed at 22 °C using a Zeiss Axioskop 2 compound microscope equipped with Nomarski DIC accessories, a Hamamatsu ORCA-ER digital camera, and Openlab image acquisition software (Improvision). Pictures were taken every 3 min for 200 min, starting 180 min after the first cell division. The time of appearance of each corpse was recorded. For each time point, 40 serial z sections at 0.5 μm/section were recorded. Recording began at 180 min after first cleavage, because the first apoptotic cell corpses appear at approximately 200 min after the first cell division [36]. Images were analyzed with ImageJ 1.40 (rsbweb.nih.gov/ij/) using the plugins Manual Tracking (rsbweb.nih.gov/ij/plugins/track/track.html) and Ome Loci (http://www.loci.wisc.edu/ome/). *p*-Values for comparisons between strains were calculated using the Wilcoxon rank-sum test.

### Quantitation of DTC defects.

Adult animals 18 h after the mid-fourth larval stage (L4) were anaesthetized and viewed as described above in “Quantitation of engulfment defects,” and gonads were visualized [85,86]. Only gonads that were completely visualized were scored. DTC migration was scored as defective when the gonad was morphologically abnormal (extra turn, two arms, or bizarre twists) or when the gonad was short or long. Gonadal length was defined as abnormal when the gonad tip was distal to the ipsilateral spermatheca (short) or distal to the contralateral spermatheca (long). The vast majority of abnormalities were in morphology rather than in length. *p*-Values for pairwise comparisons were calculated by Fisher's exact test.

### *abl-1* rescue.

P*_hsp_abl-1* and P*_hsp_abl-1(K340R)* were constructed as follows. The entire coding sequence (bp 1–3,675) of *abl-1* was synthesized by PCR from an *abl-1* cDNA template (yk1482h02, kindly provided by Y. Kohara). The PCR product was cloned into the vector pCR8/GW/TOPO using the TA cloning kit (Invitrogen). *abl-1(K340R)* was generated using the Quickchange Site-Directed Mutagenesis kit (Stratagene) from the pCR8/GW/TOPO plasmid containing the *abl-1* PCR product described above using the following primers: forward: 5′-CGACATGACTGCACAATTGCAGTGCGAGCGTTGAAGGAAGATGCAATGCC-3′; reverse: 5′-GGCATTGCATCTTCCTTCAACGCTCGCACTGCAATTGTGCAGTCATGTCG-3′. These primers created the K340R mutation. DNA sequences of PCR products were determined for accuracy and orientation. *abl-1* products were then cloned into pDEST-MB1 and pDEST-MB7 (kindly provided by M. Boxem) using the Gateway method (Invitrogen). pDEST-MB1 and pDEST-MB7 are the plasmids pPR49.78 and pPR49.83, respectively, with Gateway cassettes inserted, allowing for Gateway cloning. The P*_hsp_gfp* plasmids have been described previously [12]. P*_hsp_abl-1*, P*_hsp_abl-1(K340R)*, and P*_hsp_gfp* plasmids were injected into *ced-10(n1993); abl-1(n1963)* animals at a concentration of 20 ng/μl with a plasmid containing *myo-2::rfp* as a coinjection marker at 5 ng/μl and with 35 ng/μl of 1 Kb Plus DNA Ladder (Invitrogen) for a total concentration of 80 μg/ml per injection. The pharynges of transgenic animals were RFP-positive. Embryos were grown at 20 °C, heat shocked for 1 h at 33 °C, placed at 20 °C for up to 4 h, and then cell corpses were counted in the heads of newly hatched first larval stage (L1) animals. Two independent transgenic lines were analyzed for each transgene combination.

### Expression analysis of the *abi-1* promoter.

A PCR product containing the *abi-1* promoter (622 bp of DNA encoding the sequence between the 5′ end of the *abi-1* gene and the adjacent gene start [*B0336.5*]) followed by the *gfp* gene coding sequence was constructed as described in Wormbook (http://www.wormbook.org/chapters/www_reportergenefusions/reportergenefusions.html). The *gfp* used encodes GFP[S65C] from the vector pPD95.75 from the Fire Lab C. elegans kit (Addgene). The PCR product was injected into the gonads of *unc-76(e911)* animals at a concentration of 10 ng/μl with a plasmid containing the *unc-76* gene (p76-16B) [87] as a coinjection marker at 5 ng/μl and with 1 Kb Plus DNA Ladder (Invitrogen) at 50 ng/μl. Three independent non-Unc transgenic lines were observed and photographed using fluorescence microscopy and DIC microscopy.

### RNA interference by feeding.

Animals were fed bacteria that contained either the RNAi empty feeding vector L4440 [63] or an RNAi feeding vector with part of the *abi-1* gene, *B0336.6*, cloned into it. We obtained the *abi-1* feeding plasmid from Open Biosystems. The DNA sequence of the clone was determined to verify its accuracy. Feeding RNAi was performed as described [63,64]. Briefly, bacteria were grown in liquid culture overnight and then plated on NGM plates containing 1 mM isopropyl-D-β-thiogalactopyranoside (IPTG). Fourth-larval stage (L4) animals were placed on these plates and 24 h later were transferred to fresh plates. Progeny were tested for engulfment or DTC migration defects.

### In vitro binding.

PCR products were made from the *abl-1* cDNA template yk1482h02 and an *abi-1* cDNA template (yk1204a12). The *abl-1* products spanned bp 334–1,833 (ABL-1[112–611] and 1,818–3,675 ABL-1[606–1,224]) of yk1482h02, and the *abi-1* product (ABI-1) spanned bp 47–1,456 of yk1204a12. PCR products were cloned into the vector pCR8/GW/TOPO using the TA cloning kit (Invitrogen). The *abi-1*Δ product also spanned bp 47–1,456 of yk1204a12 but was missing bp 1,098–1,297. This construct was made using the QuickChange Site-Directed Mutagenesis kit (Stratagene) with the CR8/GW/TOPO plasmid containing the full length *abi-1* gene as a template and the following PCR primers: forward 5′-CCGACATGATCTTCCACCTCCACCAACGGGTCCTGTACGACTATGATGCTGC-3′; reverse 5′-GCAGCATCATAGTCGTACAGGACCCGTTGGTGGAGGTGGAAGATCATGTCGG-3′. DNA sequences of PCR products were determined for accuracy and orientation. *abl-1* products were then cloned into pDEST14 using the Gateway method (Invitrogen) for expression in vitro. Full-length *abi-1* and *abi-1*Δ were cloned into pDEST15 to make N-terminal glutathione S-transferase fusions using the Gateway method for expression in Escherichia coli. ABL-1 fragments were transcribed, translated, and labeled with ^35^S in vitro using the TNT T7 Quick Coupled Transcription/Translation system (Promega) according to the manufacturer. GST and GST-ABI were expressed in BL21(DE3)-RIPL cells ((BL21[DE3] Codon Plus [Stratagene]). Bacterial lysates were prepared by lysis in a French Press in the presence of protease inhibitors (Roche Applied Science). Protein binding to glutathione beads was done as follows. Glutathione beads (GE Healthcare) were washed three times in NETN (0.5% NP-40, 20 mM Tris-Cl [pH 8], 100 mM NaCl, 1 mM EDTA) and then a 1:1 mixture of beads:NETN slurry was added to soluble GST, GST-ABI, or GST-ABIΔ. The resulting mixture was incubated at 4 °C with gentle rocking for 1 h. Protein-bound beads were then washed twice with PBB (25 mM HEPES [pH 7.6], 100 mM NaCl, 5 mM MgCl_2_, 0.1 mM EDTA, 0.02% Tween-20). Binding of GST-proteins to in vitro translated proteins was done as follows. 15 μl protein-bound beads containing equivalent amounts of GST, GST-ABI, or GST-ABIΔ were added to 25 μl of a 100 μl in vitro transcription/translation reaction containing either ABL-1(112–611), ABL-1(606-1224), or Luciferase. Mixtures were diluted to 250 μl in PBB2 (25 mM HEPES [pH 7.6], 150 mM NaCl, 5 mM MgCl_2_, 0.1 mM EDTA, 0.1% Tween-20, 0.25% BSA) and incubated at 4 °C with gentle rocking for 2 h. Beads were washed three times in PBB3 (25 mM HEPES [pH 7.6], 300 mM NaCl, 5 mM MgCl_2_, 0.1 mM EDTA, 0.1% Tween-20, 0.25% BSA) with 15 min rocking at 4 °C between each wash and then washed once in PBB4 (25 mM HEPES [pH 7.6], 250 mM NaCl, 5 mM MgCl_2_, 0.1 mM EDTA, 0.1% Tween-20) followed by 15 min rocking at 4 °C. Beads were pelleted, supernatant was removed, and beads were resuspended in 2× loading dye, boiled and separated on SDS-PAGE. Gels were stained with Coomassie Blue, dried, and exposed overnight on photographic film. For quantitation of radioactivity, gels were exposed overnight on a phosphorimager screen and analyzed using a Typhoon 9400 Variable Mode Imager (Amersham Biosciences). Bands were quantified using ImageQuant 5.2 software (GE Healthcare).

## Supporting Information

Figure S1 GFP::GEX-3 Localization Is Unchanged in Embryos Lacking ABL-1 *unc-24(e138) gex-3(zu196)* and *unc-24(e138) gex-3(zu196); abl-1(ok171)* embryos containing a rescuing *gfp::gex-3* transgene were observed using a confocal microscope. (A, C, E, and G) are DIC micrographs and (B, D, F, and H) are epifluorescence micrographs. Dashed lines encircle cell corpses and their corresponding regions in the fluorescence images.
(4.23 MB PDF)

Text S1 Supplementary Text of Protocols and Results Supplementary text includes the following sections: (1) Complementation testing, mapping, and DNA sequence determination of *abl-1* alleles; (2) Determination of *abi-1* gene structure; (3) GFP::GEX-3 embryonic localization.
(45 KB DOC)

## Abbreviations

Abi: Abl interactor
DIC: differential interference contrast
DTC: distal tip cell
GEF: guanine nucleotide exchange factor
SH: Src homology
