# Supplementary figures and images for: Abl Kinase Inhibits the Engulfment of Apopotic Cells in Caenorhabditis elegans
Source: PLoS Biol. 2009 Apr 28;7(4):e1000099. doi: 10.1371/journal.pbio.1000099 (PMC2672617; doi:10.1371/journal.pbio.1000099)

***gex-3(zu196)***

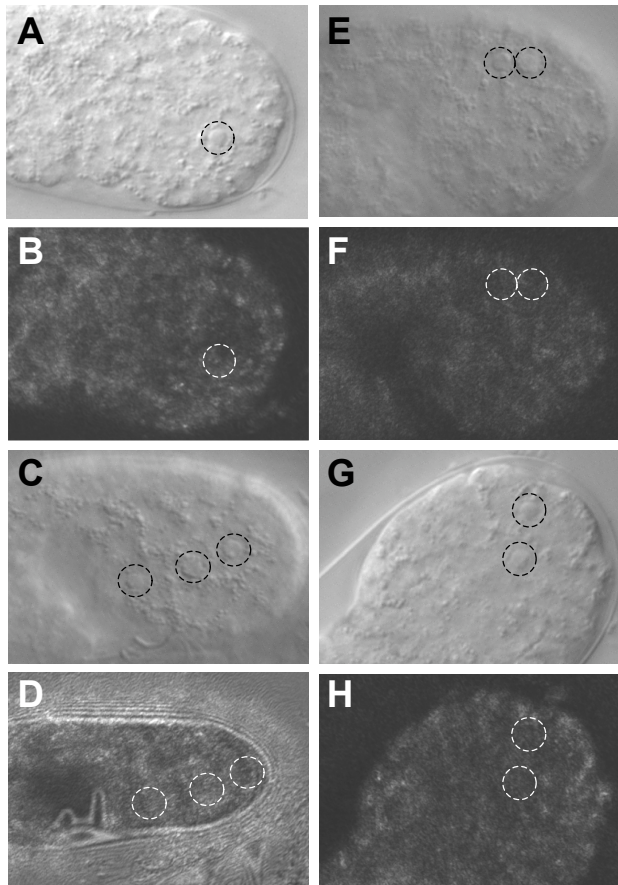

***gex-3(zu196); abl-1(ok171)***

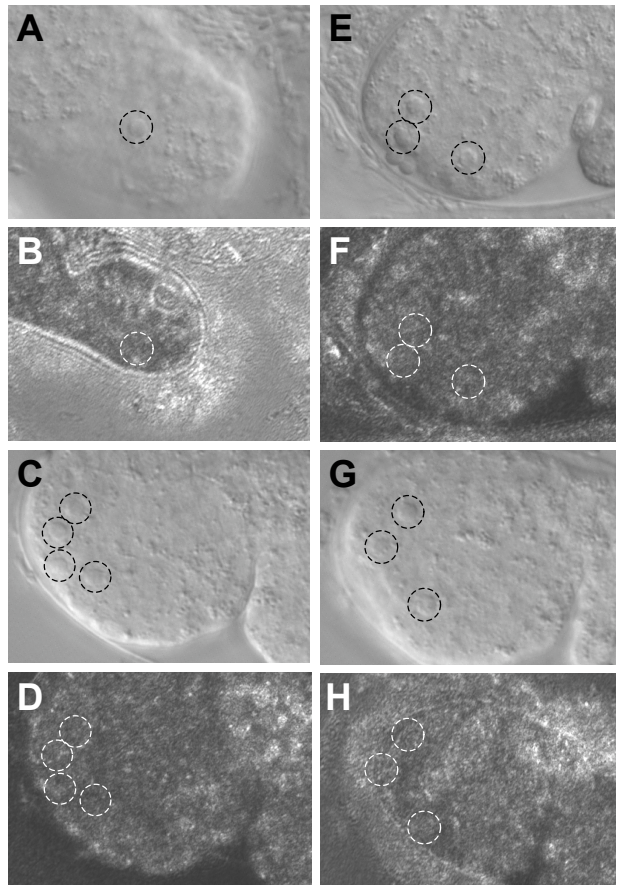

Supplement: Figure S1 — unc-24(e138) gex-3(zu196) and unc-24(e138) gex-3(zu196); abl-1(ok171) embryos containing a rescuing gfp::gex-3 transgene were observed using a confocal microscope. (A, C, E, and G) are DIC micrographs and (B, D, F, and H) are epifluorescence micrographs. Dashed lines encircle cell corpses and their corresponding regions in the fluorescence images. (4.23 MB PDF) [file pbio.1000099.sg001.pdf]
