# Supplementary material for: Abl Kinase Inhibits the Engulfment of Apopotic Cells in Caenorhabditis elegans
Source: PLoS Biol. 2009 Apr 28;7(4):e1000099. doi: 10.1371/journal.pbio.1000099 (PMC2672617; doi:10.1371/journal.pbio.1000099)
Supplement: Text S1 — Supplementary text includes the following sections: (1) Complementation testing, mapping, and DNA sequence determination of abl-1 alleles; (2) Determination of abi-1 gene structure; (3) GFP::GEX-3 embryonic localization. (45 KB DOC) [file pbio.1000099.sd001.doc]

# Supplementary Information

# Complementation testing, mapping and DNA sequence determination of *abl-1* alleles

The mutations and integrants used for mapping and complementation testing were: LGI: *dpy-5(e61)*; LGII: *bli-2(e768), juIs76[Punc-25::gfp]* [1]; LGIII: *unc-32(e189)*, *dpy-19(e1259ts)*; LGIV: *egl-20(n585)*, *bli-6(sc16)*; LGV: *rol-4(sc8), dpy-11(e224)*; LGX: *lon-2(e678).* Mutant alleles for which no citation is given were described previously [2].

To assign mutations to linkage groups, suppressed strains were mated with strains doubly mutant for *unc-34* and a visible marker located near the center of each chromosome. F_1_ Unc hermaphrodites were transferred individually to plates and allowed to lay eggs. All F_1_ progeny were Unc, demonstrating that all *abl-1* alleles have a recessive phenotype. Then F_2_ non-Unc progeny were transferred individually to plates and allowed to lay eggs. Based on the fraction of plates containing F_3_ animals in which some animals displayed the visible marker phenotype, we assigned suppressor mutations to linkage groups.

We mapped *gm332* to a small region of LG X by SNP polymorphism mapping [3]. We mated *unc-34(gm114)* animals with the CB4856 Hawaiian isolate wild-type strain five times, then assayed for the presence of Hawaiian versus Bristol markers in the center and on the ends of each chromosome. We selected one line (hereafter called *unc-34-Hw*) that carried Hawaiian markers everywhere except the left arm of V, where *unc-34* is located. Hawaiian males were mated with *unc-34-Hw* hermaphrodites, and the male progeny were then crossed with *unc-34(gm114); gm332* hermaphrodites. F_1_ Unc hermaphrodites were transferred individually to plates and allowed to lay eggs. F_2_ suppressed (non-Unc) hermaphrodites were isolated and tested for the following SNPs on LG X: snp_ZC449, snp_F45E1, snp_F11A1, snp_C23H4, snp_R01E6, snp_F23D12, snp_C33A11. Analysis after one round of mapping gave endpoints between snp_F45E1 (bp 7982357 of chromosome X) and snp_F11A1 (bp 10628472 of chromosome X). Because Abl proteins are known to oppose the function of Ena proteins [4] (UNC-34 is the *C. elegans* Ena ortholog) and *abl-1* is within this region, we suspected that these *unc-34* suppressors carried mutations in *abl-1,* and we then performed complementation tests with the deletion mutant *abl-1(ok171)*.

Two independent complementation tests were conducted by crossing *juIs76*/+; *unc-34(gm114)/+; abl-1(ok171)* hemizygous males with hermaphrodites carrying both *unc-34* and suppressor mutations*.* The F_1_ progeny were examined for suppression of the Unc phenotype. If all cross progeny were non-Unc, the mutations failed to complement. Cross progeny were identified by the presence of the *juIs76* transgene (neuronal GFP-positivity). The person scoring Unc was blinded as to which plates contained animals from particular crosses.

To molecularly characterize presumed *abl-1* mutations, we determined the sequence of PCR products from the genomic DNA of strains carrying *gm327, gm332, n1961, n1963* and *n1964*. We analyzed exons, splice junctions, the 5’ untranslated region (UTR), half of the 3’UTR and most of the introns, especially those at the 3’ end of the gene. PCR products were purified from agarose gels, and their sequences were determined. All mutations identified were confirmed by conducting a new sequencing reaction using the same genomic DNA sample.

The *abl-1* gene in *gm332* animals contained a deletion of 1400 bp from the third to the fifth intron, removing the fourth and fifth exons, which code for non-conserved regions of *abl-1*. Also, *abl-1* from these animals contained an insertion of twenty-three bp, five of which are As, while the remaining eighteen appear to be a duplication of a sequence found in the second intron of a serine protease trypsin family gene, F48E3.4. F48E3.4 is located 3.1 Mb upstream of *abl-1*. The deletion endpoints correspond to bp 21275-22696 of the M79 cosmid sequence. In addition, the mutant *abl-1* gene contains an A-to-C transversion in the third intron, four bp upstream of the beginning of the deleted sequence. If the third exon inappropriately spliced to exon six, a frame shift would result, causing a premature stop codon. Only the *abl-1E* transcript (five transcripts of *abl-1* have been identified [5]) could be unaffected by *abl-1(gm332)*, although it is possible that the deletion could affect the promoter or SL1 splicing of *abl-1E*. Suppression of the *unc-34(gm114)* Unc phenotype by *abl-1(gm332)* is as strong as *unc-34(gm114)* suppression by the deletion *abl-1(ok171),* and we predict that *gm332* is a null allele of *abl-1*.

The *abl-1* gene in *n1961* and *gm327* animals contained single missense mutations, both in exon 10, which encodes the kinase domain. *n1961* is a G-to-A transition at bp 15744 of the M79 cosmid sequence. This created a change from the glycine at position 505 to arginine. This glycine is a highly conserved amino acid that is found in almost all serine/threonine and tyrosine kinases [6]. *abl-1(gm327)* is a G-to-A transition in bp 15827 of the M79 cosmid sequence. This created a change from proline to leucine at position 477, immediately next to the substrate-recognition domain. There are other tyrosine kinases that have a leucine at this position, perhaps indicating that this is a conservative change. Consistent with that hypothesis, the suppression of the Unc phenotype seen with *gm327* is weaker than that seen with other alleles of *abl-1* including *ok171,* *gm332, n1961* and *n1963.*

*n1963* is a G-to-A transition at bp 16967 of the M79 cosmid sequence, which corresponds to the splice acceptor of exon 10. Based on suppression of the Unc phenotype, this allele is as strong as *ok171* and *gm332.*

The sequence of the *abl-1* gene of *n1964* animals was determined but no mutation was found. *n1964* failed to complement *ok171,* suggesting that *n1964* is indeed an allele of *abl-1*. The mutation in *n1964* might be further upstream in the promoter, further downstream in the 3’ UTR, or in one of the large introns near the 5’ end of the gene since these regions were not fully sequenced. Alternatively, it is possible that *n1964* is a mutation in a gene closely linked to *abl-1*.

**Determination of *abi-1* gene structure**

To define the structure of the *abi-1* transcript, we determined the sequences of the cDNA clones yk1204a12, y1170g4 and yk1680c7 (kindly provided by Y. Kohara., National Institute of Genetics, Mishima, Japan), which correspond to the gene *B0336.6* */ abi-1*. The coding sequences of yk1204a12 and yk1680c7 were identical. Translation of that sequence is shown in Figure 6A. The coding sequence of yk1170g4 had a G instead of an A at bp 218, which would result in a change from aspartic acid to glycine at amino acid 73. The 5’ sequences were identical except for yk1680c7, which was missing the last 9 bp of the SL1 sequence.

The cDNAs started with an SL1 leader sequence followed by 24 bp of 5’ noncoding sequence and the predicted translational start of the *abi-1* open reading frame. The *abi-1* open reading frame was 1410 bp followed by a 124 bp untranslated region and then a polyA tail.

# GFP::GEX-3 embryonic localization

Embryos from animals of the genotypes *unc-24(e138) gex-3(zu196)* and *unc-24(e138) gex-3(zu196); abl-1(ok171)* containing a *gfp::gex-3* rescuing transgene [7] were mounted on agarose pads and observed using a 63X objective on a Zeiss LSM510 confocal microscope.

# Supplementary References

1. Huang X, Cheng HJ, Tessier-Lavigne M, Jin Y (2002) MAX-1, a novel PH/MyTH4/FERM domain cytoplasmic protein implicated in netrin-mediated axon repulsion. Neuron 34: 563-576.

2. Riddle DL, Blumenthal T, Meyer BJ, Priess JR (1997) *C. elegans* II. Plainview, NY: Cold Spring Harbor Laboratory Press. 1222 p.

3. Wicks SR, Yeh RT, Gish WR, Waterston RH, Plasterk RH (2001) Rapid gene mapping in *Caenorhabditis elegans* using a high density polymorphism map. Nat Genet 28: 160-164.

4. Gertler FB, Doctor JS, Hoffmann FM (1990) Genetic suppression of mutations in the *Drosophila* abl proto-oncogene homolog. Science 248: 857-860.

5. Deng X, Hofmann E, Villanueva A, Hobert O, Capodieci P, et al. (2004) *Caenorhabditis elegans* ABL-1 antagonizes p53-mediated germline apoptosis after ionizing irradiation. Nat Genet 36: 906-912.

6. Hanks SK, Quinn AM, Hunter T (1988) The protein kinase family: conserved features and deduced phylogeny of the catalytic domains. Science 241: 42-52.

7. Patel F, Bernadskaya Y, Chen E, Jobanputra A, Pooladi Z, et al. (2008) The WAVE/SCAR complex promotes polarized cell movements and actin enrichment in epithelia during *C. elegans* embryogenesis. Developmental Biology 324: 297-309.
